# Non-Inferiority Study of Two Capsaicin Formulations for Painful Diabetic Neuropathy

**DOI:** 10.3390/life15101507

**Published:** 2025-09-24

**Authors:** Tamara Rodríguez Araya, Francisco Abad-Santos, José Miguel Sempere Ortells, Juan Nieto Navarro, Pablo González-López, Javier Abarca-Olivas, Elena Baño Ruiz, Carlos Iglesias-García, José Fernando Villalba García

**Affiliations:** 1 Primary Chronic Pain and Fibromyalgia Unit, Hospital Clínic in Barcelona, 170 Villarroel Street, 08036 Barcelona, Spain; tlrodriguez@clinic.cat; 2 Clinical Pharmacology Department, Hospital Universitario de La Princesa, Faculty of Medicine, Instituto de Investigación Sanitaria La Princesa (IP), Universidad Autonóma de Madrid (UAM), 28006 Madrid, Spain; francisco.abad@salud.madrid.org; 3 Centro de Investigación Biomédica en Red de Enfermedades Hepáticas y Digestivas (CIBERehd), Instituto de Salud Carlos III, 28029 Madrid, Spain; 4 Department of Biotechnology, University of Alicante, San Vicente del Raspeig Road, San Vicente del Raspeig, 03690 Alicante, Spain; josemiguel@ua.es (J.M.S.O.); villalbajf@gmail.com (J.F.V.G.); 5 Department of Neurosurgery, Miguel Hernández University of Elche, University Avenue, 03202 Elche, Spain; jnieto@umh.es; 6 Department of Neurosurgery, University General Hospital of Alicante, 03010 Alicante, Spain; pablo.gonzalezl@umh.es (P.G.-L.); jabarcaolivas@gmail.com (J.A.-O.); ebaruiz@gmail.com (E.B.R.); 7 Clinical Partner & Innovation, S.L. 49 Princesa Street, 28008 Madrid, Spain

**Keywords:** capsaicin, painful diabetic peripheral neuropathy, crossover study, non-inferiority clinical trial, safety

## Abstract

While capsaicin topical formulations are established treatments, conventional creams using this substance have limited application due to handling-related adverse events. This study aimed to demonstrate that the efficacy of a novel 0.75 mg/g capsaicin roll-on solution is non-inferior to the approved 0.75 mg/g cream in patients with painful diabetic neuropathy (PDN). In total, 160 patients were randomized to receive either the roll-on or the cream, applied four times daily for 8 weeks, followed by a 4-week washout period and crossover to the alternate treatment. The primary endpoint was pain intensity (numerical rating scale), with secondary endpoints including quality of life (EQ-5D) and safety. Both groups showed significant reductions in pain, with no statistically significant differences in absolute (*p* = 0.115) or relative (*p* = 0.157) pain reduction. Non-inferiority was confirmed with a 95% CI for the difference in mean pain reduction [−0.86–0.07], remaining within the pre-specified margin (1.0 unit). Quality of life improved in both groups, with no significant differences (*p* = 0.266). The incidence of adverse events was low and predominantly mild, with no significant differences between groups (*p* = 0.424) and a favorable trend for the roll-on formulation. The roll-on capsaicin formulation demonstrated non-inferiority in efficacy and safety compared with the conventional cream formulation.

## 1. Introduction

Neuropathic pain is defined as pain arising from a lesion or disease affecting the somatosensory nervous system. Such pain is a clinical descriptor rather than a definitive diagnosis. Confirmation requires not only pain recognition but also understanding of the underlying etiology, supported by diagnostic studies such as neurophysiological tests, biopsies, or imaging. The anatomical distribution of pain must match the corresponding somatosensory dysfunction. Common causes include peripheral nerve injury or entrapment, central nervous system disorders, and systemic conditions such as diabetes mellitus (DM), viral infections (e.g., postherpetic neuralgia), multiple sclerosis, cancer, and neurotoxic exposure (e.g., chemotherapy) [1,2,3,4]. The estimated prevalence of neuropathic pain in the general population ranges from 0.9% to 17.9%, significantly impacting quality of life (QoL) [5]. Moreover, the incidence of such pain is expected to continue to rise [6].

Due to differences in patients’ subjective perception, among other causes, the clinical severity of neuropathic pain does not consistently correlate with lesion extent. Patients frequently describe this pain as stabbing, electric shock-like, burning, or searing. Sensory deficits such as numbness, tingling, and prickling are common. Pain may occur spontaneously or be triggered by mild stimuli (hyperalgesia, allodynia) and may include autonomic signs such as changes in skin color, temperature, sweating, or edema [7,8].

DM represents the most prevalent endocrine disorder, with an incidence of 1–2%, among which type 2 DM is the most common form [9,10,11]. Increased life expectancy in diabetics is linked to more chronic complications [12,13,14]. Diabetic neuropathy (DN) stands out among microvascular complications and involves both metabolic and vascular factors in its pathogenesis [15,16]. DN represents a spectrum of neuropathic conditions with clinical manifestations ranging from peripheral neuropathy to various forms of autonomic dysfunction, including cardiovascular, gastrointestinal, and urogenital issues. The baseline prevalence of PDN in newly diagnosed or early Type 2 DM may be as high as 10–20%, with progression to rates exceeding 50% after 10 or more years. Individuals with PDN may experience severe pain, loss of sensation, impaired balance, falls, ulcers and amputations, all leading to reduced quality of life [17]. Painful diabetic peripheral neuropathy (PDPN) is a leading cause of chronic neuropathic pain, affecting 0.8% of the general population and up to 26.4% of patients with type 2 DM. This pain’s most common manifestation is distal symmetric sensory polyneuropathy, typically causing pain and paresthesia in the lower limbs, described as burning, stabbing, or lancinating [15,16]. PDPN often involves dysesthesias, thermal sensitivity changes, skin discoloration, abnormal sweating, hyperalgesia, allodynia, and sensory loss, increasing the risk of unperceived injuries, potentially leading to infection, ulceration, limb amputation, or death [12,13,18,19].

Neuropathic pain management remains a clinical challenge. First-line pharmacological treatments—tricyclic antidepressants, serotonin-noradrenaline reuptake inhibitors (SNRIs), and gabapentinoids—yield suboptimal outcomes [6,20]. A personalized, multidisciplinary approach is therefore recommended, with the early introduction of single or combined medications and escalation to interventional therapies when necessary [2,13,21]. Second-line treatments include tramadol, topical capsaicin, and lidocaine patches; third-line therapies include botulinum toxin and opioids, though classifications vary among guidelines [20,22,23].

Capsaicin has been marketed in Spain since 1995 and is authorized for moderate-to-severe PDPN, particularly in patients unresponsive to first-line agents or as adjunctive therapy. This substance is the primary pungent compound in chili peppers of the Capsicum genus and acts on nociceptive neurons (C and Aδ fibers) via Transient Receptor Potential Vanilloid 1 (TRPV1). Activated TRPV1 receptors release neuropeptides such as calcitonin gene-related peptide (CGRP) and substance P. Repeated or high-concentration exposure to capsaicin leads to desensitization [24,25], pain, and itching, while other sensory functions remain intact.

Several controlled clinical trials [26,27,28,29] involving 588 patients have assessed the efficacy of 0.075% topical capsaicin applied for PDPN four times daily over 8 weeks. A 2009 Cochrane review concluded that repeated applications of low-dose capsaicin (0.075%) and high-dose capsaicin patches may provide pain relief in patients with neuropathic pain [30,31].

One main limitation of topical capsaicin creams relates to handling. Accidental contact with the eyes or mucous membranes can cause intense stinging or burning, compromising adherence. The new roll-on formulation prevents direct contact by forming a solid, transparent film on the skin, allowing safe handling and gradual absorption, minimizing the risk of accidental transfer. This study aims to evaluate the efficacy of a roll-on topical capsaicin device compared with a conventional cream formulation, both indicated for the treatment of painful diabetic neuropathy.

## 2. Materials and Methods

### 2.1. Study Design

**Figure 1:**
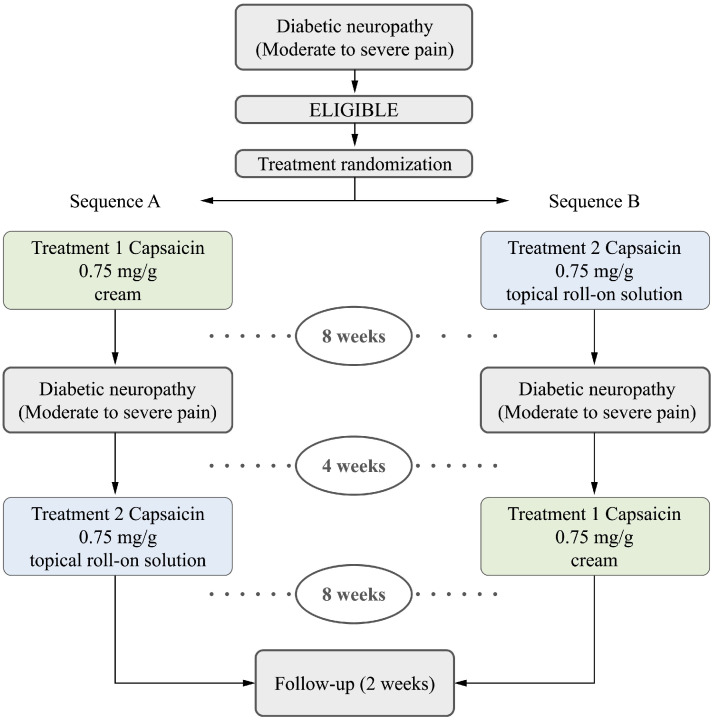
Study flow and treatment regimen scheme.

This study was conducted as a randomized, open-label, multicenter, crossover, non-inferiority clinical trial across primary healthcare centers in Spain. This research aimed to compare two topical capsaicin formulations for the relief of moderate-to-severe pain in human participants with painful diabetic neuropathy. As illustrated in Figure 1, eligible patients were randomized to receive either the experimental (capsaicin 0.75 mg/g topical solution applied via roll-on device) or comparator treatment (capsaicin 0.75 mg/g cream [Arafarmadol^®^ 0.75 mg/g cream; Arafarma Group, S.A., Marchamalo (Guadalajara), Spain]), administered four times daily over an 8-week period. The experimental solution was applied by spreading a thin layer over the affected area using the roll-on device without rubbing, performing 1 to 3 passes three to four times daily for 8 weeks.

Upon completion of the first treatment period, a 4-week washout phase was implemented. Patients then crossed over to the alternative treatment for an additional 8 weeks, followed by a 2-week follow-up phase after the second treatment period. As illustrated in Figure 1, patients were randomized to receive treatment sequence A (comparator drug followed by experimental) or sequence B (experimental drug followed by comparator). The total study duration was 22 weeks.

### 2.2. Outcomes

The primary objective was to demonstrate the non-inferiority, in terms of efficacy, of a new topical capsaicin 0.75 mg/g solution applied via roll-on compared with the authorized cream formulation (0.75 mg/g cream). The primary efficacy endpoint was a change in pain intensity, assessed using a numerical pain rating scale. Arafarma Group S.A. supplied both formulations in sufficient quantities for all patients enrolled in the study. Secondary objectives included an evaluation and comparison of safety, assessment of quality of life, evaluation of treatment adherence, and patient satisfaction, using a self-administered questionnaire to compare outcomes between the two treatments.

### 2.3. Patient Involvement

Patient involvement in this study was limited to their participation as study subjects.

### 2.4. Eligibility Criteria

A screening visit was conducted to evaluate the inclusion and exclusion criteria (Table 1). Prior to treatment initiation, a comprehensive assessment was performed to confirm patient eligibility. This assessment included a detailed medical history, review of concomitant medications, physical examination, lower limb evaluation, identification of painful sites, dermatological assessment of affected areas, baseline pain assessment using a numerical scale, and a quality-of-life questionnaire. Regardless of the sequence assigned (A or B), each patient received both formulations for 8 weeks each.

### 2.5. Study Procedures

Ongoing treatments for diabetes management, antidepressants, and anticonvulsants were permitted. Paracetamol or ibuprofen could be prescribed in cases of moderate burning sensations post-application or inadequate pain relief in addition to a stable dose of pain medication maintained for at least one month before enrolment. Patients were required to remain on a stable analgesic regimen and dosage throughout the study and follow-up. The use of local anesthetics was not permitted.

Study treatment was discontinued if pain persisted or worsened after the first two weeks, if pain resolved but recurred shortly thereafter, or if excessive irritation occurred.

The primary outcome was the evaluation of pain intensity and improvement using a numerical rating scale from 0 (no pain) to 10 (unbearable pain). Secondary outcomes included a safety assessment and evaluation of quality of life using the European Quality of Life Questionnaire (EQ-5D).

Pain intensity and improvement, dermatological assessments, evaluation of the painful area, concomitant medication review, and adverse event reporting were conducted at 2, 4, 6, and 8 weeks after initiation of each treatment. Additionally, a physical examination and lower limb assessment (DN4 test) were conducted at week 4. Quality of life was assessed using the EQ-5D prior to treatment initiation and after each 8-week treatment period. Final assessments included a physical examination, lower limb assessment, pain evaluation, dermatological assessment, documentation of adverse events, and review of concomitant medication.

Physical examination included several variables: Body Mass Index (BMI), Systolic Blood Pressure (SBP), and Diastolic Blood Pressure (DBP). BP was measured noninvasively via auscultation of the brachial artery with a stethoscope to detect the appearance and muffling or disappearance of Korotkoff sounds, which represent SBP and DBP, respectively, in a seated position with the cuff located at the level of the patient’s right atrium. The patient was seated for 3–5 min without talking or moving before recording the first BP reading. The patient avoided caffeine, exercise, and smoking for at least 30 min before measurement and emptied his/her bladder. Devices were calibrated periodically. On average, 2 readings obtained on two occasions were used to estimate the individual’s BP. BMI was calculated using the weight and height (weight divided by height squared). BP and BMI were obtained at visits 0 and 6 and at 4 weeks of treatment with each of the formulations (visits 2 and 8).

### 2.6. Sample Size Calculations

Sample size calculation assumed a 40–60% reduction in pain intensity after 8 weeks of capsaicin treatment [28,32,33,34,35]. With a baseline pain score of approximately 6, a mean reduction of 3 units was expected. Assuming a standard deviation of 2.0 units for pain reduction and a non-inferiority margin of 1.0 units, a sample size of 60 patients per group would achieve over 80% statistical power at a 5% significance level. To account for an anticipated 30% dropout rate, 80 patients were planned for each group.

### 2.7. Randomization

Randomization was performed in blocks of eight patients (two blocks of four) using a random number table [36].

### 2.8. Statistical Analysis

The primary efficacy analysis was performed on the intention-to-treat (ITT) population, defined as all random patients analyzed according to their assigned treatment. Additionally, pain reduction was evaluated in the per-protocol (PP) population, consisting of patients without major protocol deviations. The safety population comprised all patients who received at least one dose of the study treatment. In cases of missing data, the last observation carried forward (LOCF) method was applied.

The primary endpoint was a reduction in pain intensity on the Visual Analog Scale (VAS) for pain after 8 weeks of treatment with each drug. Since the treatment received during the first treatment period can influence responses during the second period, only the first 8 weeks of treatment were considered for the non-inferiority analysis. The difference in efficacy between the two treatments was then calculated (pain reduction with the cream minus pain reduction with the roll-on), and the 95% confidence interval (CI) for this difference was obtained. If the CI did not exceed the non-inferiority limit (1.0), it was concluded that the new formulation was not inferior to the currently marketed formulation. The remaining variables were then analyzed, considering both treatment periods and comparing the response of each patient to each of the treatments in a paired analysis. Corresponding descriptive summaries of the demographic and baseline characteristics are presented. Counts and proportions are also presented for categorical variables along with descriptive statistics for continuous variables. Analysis of variance was then used to determine which demographic variables would require further assessment. These variables included investigational site, treatment sequence, sex, age, type of diabetes, duration of painful neuropathy, and pain severity at the baseline visit. An analysis of therapeutic responses to the capsaicin topical roll-on solution or cream was performed for each of the response variables after adjusting or controlling for other significant variables. Demographic data were then analyzed using Fisher’s exact test to compare the capsaicin roll-on topical solution and capsaicin cream groups with respect to sex and diabetes type. A T-test was used to compare age, diabetes duration, and neuropathy duration. Physical global assessment, intensity VAS, and improvement VAS were analyzed using analysis of variance, adjusting for the recruiting center. All tests were one-tailed at a significance level of 0.05.

Continuous variables were summarized using measures of central tendency (mean and/or median) and dispersion (standard deviation and range), while categorical variables were reported as percentages. Where applicable, 95% confidence intervals were provided for estimates.

### 2.9. Ethical Procedures

This study was conducted in accordance with the Declaration of Helsinki, the International Conference on Harmonisation Good Clinical Practice (ICH-GCP) guidelines, and applicable Spanish regulations. Ethical approval (1631A) was obtained from the Ethics Committee of Hospital Universitario de la Princesa, Madrid. Written informed consent was obtained from all participants prior to enrolment. ClinicalTrials.gov registration: NCT05029297, https://clinicaltrials.gov/ (accessed on 14 July 2025).

The CONSORT 2025 checklist reporting guidelines were used to write this manuscript [37].

## 3. Results

### 3.1. Population

**Figure 2:**
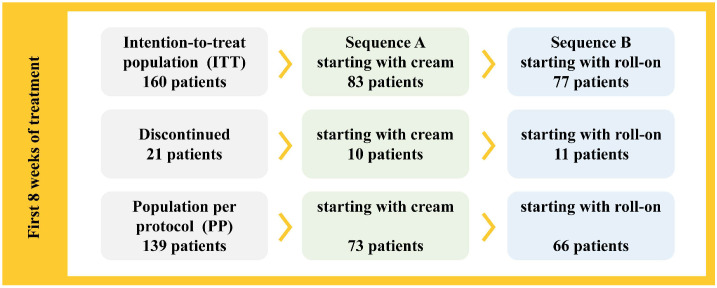
Population groups.

A total of 160 patients with PDPN initiated treatment with two topical capsaicin formulations for relief of moderate to severe pain in a crossover design. Of these, 83 patients began with the cream formulation and 77 with the roll-on solution (Figure 2). Females accounted for 46.9% of the sample, with no statistically significant differences observed between the groups in terms of sex distribution (male/female) (*p* = 0.774). The mean age of participants was 67.7 years (SD = 12.1), with no significant differences between groups (*p* = 0.163). Similarly, no significant differences were found in the ethnic distribution (*p* = 1.000), physical examination findings (Table 2), or review of systems based on medical history (*p* > 0.05).

Patient flow through the study is summarized in Table 3. The discontinuation rate was 13.13% during the first treatment phase and 18.13% when both phases were considered, remaining below the pre-established acceptable threshold of 30%. Consequently, to analyze the primary endpoint, the intention-to-treat (ITT) population included all 160 patients (cream group: 83; roll-on group: 77), while the per-protocol (PP) population comprised 139 patients (cream group: 73; roll-on group: 66). The 21 patients excluded from the PP population (10 from the cream group and 11 from the roll-on group) demonstrated poor adherence during the first treatment phase. Reasons for discontinuation by treatment group are provided in Table 4.

### 3.2. Efficacy

**Figure 3:**
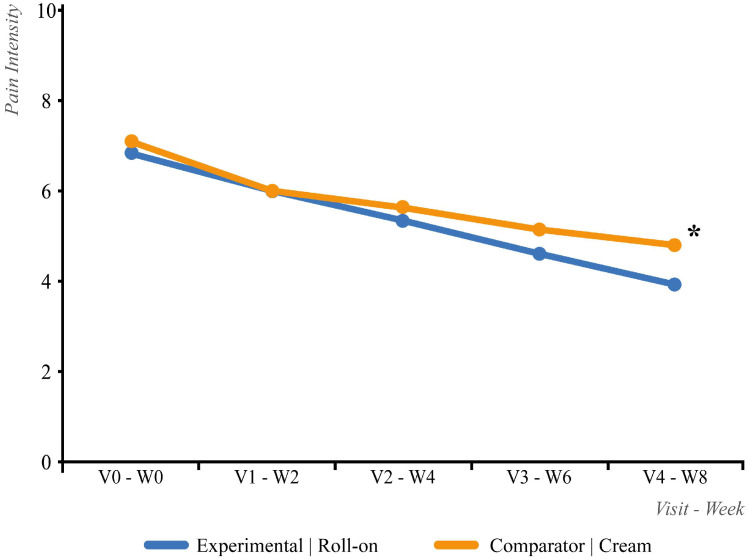
Evolution of the numerical pain intensity scale score 8 weeks after the first ITT treatment sequence. * *p* < 0.05.

Efficacy was assessed in the ITT population. Figure 3 presents the results for the first treatment sequence.

The primary variable of interest, pain intensity (measured via a visual analogue scale), improved over the first 8-week treatment phase, decreasing from 6.9 to 3.9 in the roll-on group and from 7.1 to 4.8 in the cream group. Visit-by-visit comparisons revealed no statistically significant differences between groups at baseline/week 0 (|t| = 1.05; *p* = 0.294), week 2 (|t| = 0.03; *p* = 0.973), week 4 (|t| = 0.68; *p* = 0.497), or week 6 (|t| = 1.91; *p* = 0.058). However, by week 8 (visit 4), the roll-on group exhibited significantly lower pain scores (|t| = 2.53; *p* = 0.013) (Figure 3).

In absolute terms (baseline-to-endpoint within each group), pain intensity significantly decreased in both groups: roll-on (|t| = 10.33; *p* < 0.001) and cream (|t| = 10.91; *p* < 0.001). No statistically significant difference was observed between groups for the absolute change in pain VAS scores (|t| = 1.59; *p* = 0.115). The 95% confidence interval for the mean difference was CI [−1.19–0.13], which did not exceed the pre-specified non-inferiority margin of 1.0. Therefore, the experimental treatment was considered non-inferior to the comparator.

In relative terms (percentage change from baseline), both groups also showed a significant reduction in pain intensity: roll-on (|t| = 11.29; *p* < 0.001) and cream (|t| = 10.86; *p* < 0.001). No significant difference was detected between groups regarding the relative change (|t| = 1.42; *p* = 0.157).

When defining a “responder” as a patient achieving at least a 30% reduction in pain from baseline, no significant difference in “responder” rates was observed between groups (*p* = 0.980).

An analysis of variance (ANOVA) was performed to assess potential sequence effects. The homogeneity of treatment sequences was confirmed in terms of baseline characteristics, including age (|t| = 1.40; *p* = 0.163), sex (χ^2^ = 0.08; *p* = 0.774), type of diabetes (*p* = 0.523), diabetes duration (|t| = 0.03; *p* = 0.976), BMI (|t| = 0.43; *p* = 0.665), baseline pain score (|t| = 1.05; *p* = 0.294), quality of life (|t| = 1.32; *p* = 0.188), affected area (χ^2^ = 0.73; *p* = 0.867), DN4 score (|t| = 0.59; *p* = 0.558), and neuropathic pain duration (|t| = 0.02; *p* = 0.984). Consequently, none of these variables were included as covariates in the model. ANOVA for the crossover design demonstrated no significant effect of the treatment sequence on final pain scores at week 8 (F = 1.27; *p* = 0.261).

**Figure 4:**
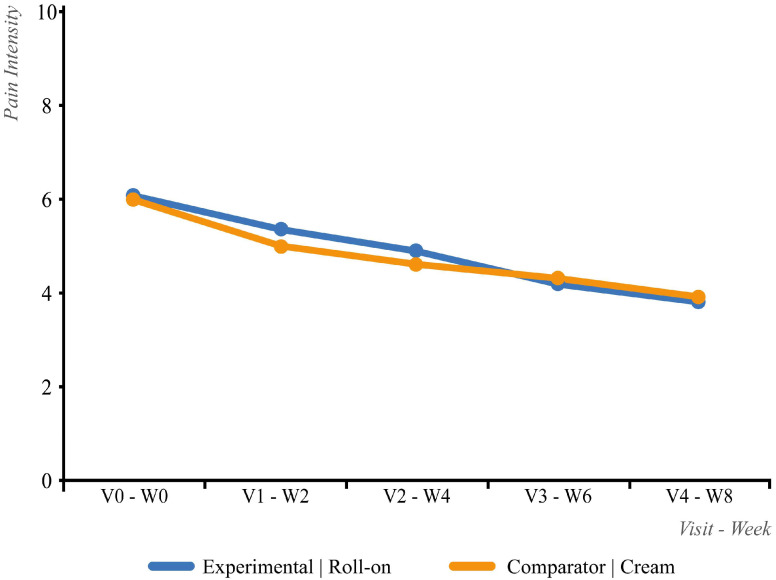
Evolution of the numerical pain intensity scale score in both IT treatment sequences.

When both treatment sequences were considered (Table 5 and Figure 4), no significant differences were observed between groups at baseline (|t| = 0.94; *p* = 0.351), week 2 (|t| = 0.59; *p* = 0.554), week 4 (|t| = 0.72; *p* = 0.473), week 6 (|t| = 0.31; *p* = 0.758), or week 8 (|t| = 0.68; *p* = 0.499) (Figure 4). The 95% CI for the difference in the mean change between treatments was [−0.86–0.07], remaining within the non-inferiority margin of 1.0 and confirming the non-inferiority of the experimental treatment. When considering the last available visit for each treatment, no significant differences were observed between groups (|t| = 0.78; *p* = 0.435). “Responder” rates were also comparable (*p* = 1.000).

**Figure 5:**
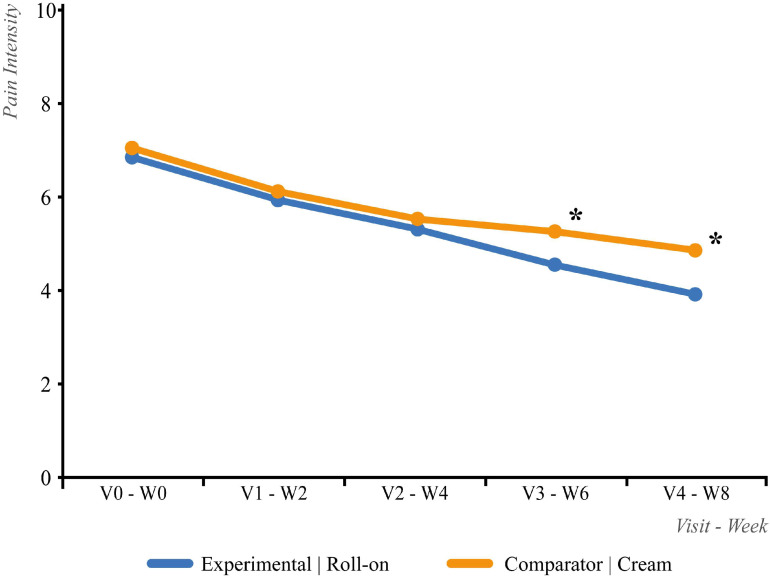
Evolution of the numerical pain intensity scale score 8 weeks after the first PP treatment sequence. * *p* < 0.05.

In the PP population analysis (Figure 5), pain intensity scores decreased significantly in both the experimental (|t| = 10.22; *p* < 0.001) and comparator (|t| = 10.94; *p* < 0.001) groups. The absolute reduction in pain was significantly greater in the experimental group than in the comparator group (−3.0 vs. −2.2 points, respectively; |t| = 2.04; *p* = 0.044). The 95% confidence interval for the difference in means was [−1.42–−0.02], remaining within the non-inferiority threshold (1.0) and confirming the non-inferiority of the experimental formulation.

A full model ANOVA revealed no statistically significant variability attributable to the treatment sequence (F = 3.70; *p* = 0.056). In absolute terms, pain scores declined significantly during the observation period in both the experimental (|t| = 11.15; *p* < 0.001) and comparator (|t| = 12.48; *p* < 0.001) groups. No statistically significant differences were observed between groups in terms of absolute score changes (|t| = 1.52; *p* = 0.132). The 95% CI for the mean difference between treatments was [−0.86–0.11], remaining within the non-inferiority margin and thus reaffirming that the experimental treatment was not inferior to the comparator.

### 3.3. Quality of Life (EQ-5D)

Among the IT population, in absolute terms (change from the baseline to final score), quality of life (QoL) improved significantly during the observation period in patients who initially received the experimental treatment (|t| = 3.52; *p* = 0.001) and among those who received the comparator (|t| = 2.94; *p* = 0.005). No significant differences were observed between treatment groups for absolute changes in QoL (|t| = 1.18; *p* = 0.266). The 95% confidence interval for the mean difference was [−2.58–9.26].

A crossover design analysis of variance (ANOVA) was performed to assess the effect of the treatment sequence on the final QoL evaluation (visit 11). The ANOVA showed that the model was not sufficiently sensitive to discriminate against the variability between treatment sequences (F = 1.61; *p* = 0.206). As no sequence effect was detected, the two treatments were compared regardless of administration order.

In absolute terms (change from the baseline to final score), QoL improved significantly during the observation period in both the experimental treatment (|t| = 5.26; *p* < 0.001) and comparator groups (|t| = 5.25; *p* < 0.001). No significant differences were found between treatment groups for absolute change in QoL (|t| = 1.10; *p* = 0.273).

Additionally, no differences were detected between groups when analyzing the PP population. In absolute terms (change from the baseline to final score), QoL improved significantly during the observation period for patients initially treated with the experimental formulation (|t| = 3.52; *p* = 0.001) and for those treated with the comparator (|t| = 2.94; *p* = 0.005). No significant differences were observed between groups for absolute change in QoL (|t| = 1.18; *p* = 0.266). The 95% confidence interval for the mean difference was [−2.58–9.26].

### 3.4. Safety

Overall, 23.8% of patients experienced adverse events (AEs), with 14.4% occurring during administration of the experimental treatment and 17.5% during administration of the comparator (Table 6). No statistically significant differences were found between treatments (*p* = 0.424), although a favorable trend towards the experimental treatment was observed. Treatment-associated AEs are detailed in Table 7. Many AEs were mild in nature.

## 4. Discussion

In patients with PDPN, the experimental roll-on formulation of capsaicin at a concentration of 0.75 mg/g demonstrated non-inferiority compared with the standard cream formulation at the same concentration during the initial 8-week treatment period. This outcome was consistently observed across both ITT and PP populations and among the pooled analysis of participants from both treatment sequences, in terms of the primary endpoint and pain intensity measured by the numeric rating scale.

In the ITT analysis, no statistically significant differences were observed in favor of the cream over the roll-on in terms of the magnitude of pain reduction during the first treatment sequence, both in absolute terms (change from baseline to endpoint) and relative terms (percentage change). However, the PP analysis revealed a statistically significant greater absolute reduction in pain scores in favor of the experimental treatment. In both groups, the pain reduction exceeded two points, a threshold commonly regarded as clinically significant [38,39,40].

The statistical analysis conducted in both ITT and PP populations was intended to enhance the robustness and reliability of the findings. One inherent limitation of randomized controlled trials is the potential for protocol deviations or treatment discontinuation, which may result in data discontinuation. An alternative approach to mitigate this risk is an “intention-to-treat” analysis, widely regarded as a conservative but comprehensive method that better reflects real-world clinical practice. In a study by Simpson DM et al., 2017 [41], which demonstrated statistically significant efficacy of an 8% capsaicin patch versus placebo in painful diabetic neuropathy, both ITT and additional PP analyses were performed. PP results supported the ITT findings, demonstrating efficacy from as early as two weeks into treatment.

The assumptions made during the sample size calculation were consistent with the results obtained. Importantly, despite anticipating a potential 30% discontinuation rate, the final discontinuation was only 13.13% after the first phase and 18.13% after the second, thereby preserving the statistical power of the study.

In terms of the secondary efficacy variable, quality of life, significant improvements were observed in both treatment arms over the study period, with no statistically significant differences between the two formulations.

Regarding safety, the adverse effects of topical capsaicin cream at 0.75 mg/g are well documented. The most common adverse events include burning or stinging sensations on the skin (very frequent, ≥1/10 patients), as well as ocular irritation (tearing) and respiratory symptoms (coughing and sneezing) classified as frequent (≥1/100 to <1/10 patients). Accidental contact with the eyes or mucosa after handling the cream remains the most limiting adverse effect. Current instructions recommend thorough hand washing with cold water and soap after application and advise against applying the cream near the eyes or mucous membranes unless specifically instructed by a physician.

The roll-on formulation employs a novel vehicle system that eliminates direct manual contact and offers a potential safety advantage by minimizing the risk of accidental mucosal exposure. This formulation may, in turn, contribute to improving therapeutic adherence. Although no statistically significant differences in safety profiles were observed between treatment groups under the controlled conditions of this study, a lower incidence of capsaicin-related adverse events is anticipated in routine clinical practice with the roll-on formulation.

Apart from the novelty of incorporating a new formulation of capsaicin, this study has several limitations. For example, the application method of the product (roll-on or cream) prevented the study from being blinded, and no multivariate model could be obtained to explain the patient’s responses after the comparator treatment. A larger study or specific patient populations could help identify patient groups with different responses to both treatments and their safety profiles. While the trial design did not confirm superior efficacy, it opens the possibility for future studies, beyond non-inferiority designs, that may detect significant statistical differences in favor of the experimental treatment. Most patients in the study presented good therapeutic adherence to both treatments, possibly due to the clinical follow-up performed during the study. An observational study with real-world data in the general population could be conducted to assess treatment adherence alongside reported adverse events in national pharmacovigilance systems for such formulations. Another limitation is the concentration of capsaicin (0.75 mg/g) used for this study. The product’s marketed dose is intended for outpatient topical treatments administered in an ambulatory setting. Therefore, the results of this study cannot be extrapolated to other capsaicin concentrations, such as 8%, which must be administered with special precautions in a clinic or hospital.

## 5. Conclusions

This study demonstrated that the novel roll-on solution containing capsaicin 0.75 mg/g is non-inferior in efficacy to the currently authorized cream formulation. Additionally, the roll-on delivery system yielded a safety profile comparable to that of other available treatments for patients with PDN. Furthermore, the user-friendly application of this treatment may contribute to improved tolerability, particularly when compared with cream formulations. This potential advantage could translate into greater adherence in real-world settings, beyond the controlled conditions of a clinical trial.

## Figures and Tables

**Table 1:** Inclusion and exclusion criteria.

**Inclusion Criteria:**
1. Written or witnessed oral informed consent obtained prior to protocol-specific procedures.
2. Male or non-pregnant, non-lactating female ≥18 years of age.
3. Patients diagnosed with type I or II diabetes mellitus, treated or untreated.
4. Patients diagnosed with painful diabetic peripheral neuropathy, treated or untreated.
5. Painful diabetic neuropathy of at least 3 months’ duration with moderate to severe pain ≥4 on the visual analogue pain scale during the screening phase.
6. Pain must have been experienced daily, must have interfered with daily activities or sleep, and must not be attributable to psychological causes.
7. Stabilization on pain medication for at least one month prior to starting treatment. The patient must be prepared to remain on the same pain medications at the same doses as those before baseline throughout the study and during the follow-up phase (24 weeks).
8. Intact, non-irritated, dry skin in the painful areas to be treated.
9. Patients able to participate in the trial.
**Exclusion Criteria:**
1. Allergic reactions to capsaicin.
2. Patients with neuropathic pain of an etiology other than diabetes.
3. Patients with peripheral ischemic pain due to diabetic arterial disease.
4. Patients with unstable glycemic control (glycated hemoglobin ≥ 10.5%).
5. Amputation of any part of the lower extremity.
6. Surgery scheduled during the clinical trial.
7. Mild painful diabetic neuropathy (<4 VAS).
8. Other serious conditions:
Documented congestive heart failure or systolic dysfunction (LVEF ≤ 50%).
Previous history of myocardial infarction within 6 months prior to inclusion.
Uncontrolled hypertension (160/110 mmHg maximum)
Uncontrolled high-risk arrhythmias.
Significant neurological or psychiatric disorders, including psychotic disorders or dementia, that prevent patients from understanding and providing informed consent.
Active, uncontrolled infection.
9. Use of other topical pain medications on the painful areas.
10. History or current substance abuse problem.
11. Pregnant or breastfeeding women. Women of childbearing potential must use effective contraception.
12. Participation in another clinical trial with any non-marketed investigational drug within 90 days prior to enrollment.

**Table 2:** Physical examination.

Variables and Values	Sequence A	Sequence B	Total
BMI (Kg/m^2^), mean ± SD	29.8 ± 4.3	30.2 ± 6.0	30.0 ± 5.2
SBP (mmHg), mean ± SD	134.2 ± 14.2	132.7 ± 13.7	133.5 ± 13.9
DBP (mmHg), mean ± SD	75.0 ± 10.1	74.4 ± 9.1	74.7 ± 9.6

BMI, Body Mass Index; SD, standard deviation; SBP, Systolic Blood Pressure; DBP, Diastolic Blood Pressure.

**Table 3:** Patient flow according to the evolution of visits.

Period	Visit/Week	Cream	Roll-On	Total
First stage	V0/W0	83	77	160
	V1/W2	80	74	154
	V2/W4	77	69	146
	V3/W6	73	69	142
	V4/W8	73	66	139
Second stage	V6/W12	72	66	138
	V7/W14	72	66	138
	V8/W16	68	62	130
	V9/W18	67	64	131
	V10/W20	67	64	131

**Table 4:** Reasons for discontinuation.

Reasons for Discontinuation	Start with		Global	
	Cream	Roll-On	n	%
Adverse Event	2	3	5	44.83%
Absence of symptoms	1	2	3	10.35%
Personal reasons	2	4	6	20.69%
Other pathologies	1	1	2	6.90%
Loss to follow-up	4	1	5	17.24%
Total	10	11	21	100.00%

**Table 5:** Patients with data available from both the comparator and experimental treatments, with no sequence effect.

Visits	Patients with Cream Treatment			Patients with Roll-On Treatment		
	First Stage	Second Stage	Total	First Stage	Second Stage	Total
V0 + V6	83	66	149	77	72	149
V1 + V7	80	66	146	74	72	146
V2 + V8	77	62	139	69	68	137
V3 + V9	73	64	137	69	67	136
V4 + V10	73	64	137	66	67	133

**Table 6:** Adverse events (AEs), patients.

AE (No/Yes)	Experimental (Roll-On)	Comparator (Cream)	Total
No	137 (85.6%)	132 (82.5%)	122 (76.3%)
Yes	23 (14.4%)	28 (17.5%)	38 (23.8%)
Total	160 (100.0%)	160 (100%)	160 (100.0%)

**Table 7:** Adverse events (AE), descriptions.

AE Description	Experimental (Roll-On)	Comparator (Cream)	Total
Erythema	13 (8.1%)	14 (8.8%)	27 (16.9%)
Itching/Stinging	4 (2.5%)	5 (3.1%)	9 (5.6%)
Burning	3 (1.9%)	4 (2.5%)	7 (4.4%)
Pruritus	2 (1.3%)	0	2 (1.3%)
Increasing Pain	1 (0.6%)	1 (0.6%)	2 (1.3%)
Edema	1 (0.6%)	1 (0.6%)	2 (1.3%)
Pharyngeal Irritation	1 (0.6%)	1 (0.6%)	2 (1.3%)
Worse Neuropathic Pain	1 (0.6%)	0	1 (0.6%)
Sneezing	1 (0.6%)	0	1 (0.6%)
Papules	1 (0.6%)	0	1 (0.6%)
Runny Nose	1 (0.6%)	0	1 (0.6%)
Urticaria	1 (0.6%)	0	1 (0.6%)
Bronchitis	0	1 (0.6%)	1 (0.6%)
Intolerance to Study Drug	0	1 (0.6%)	1 (0.6%)
Secondary Lesion	0	1 (0.6%)	1 (0.6%)
Rhinitis	0	1 (0.6%)	1 (0.6%)
Cough	0	1 (0.6%)	1 (0.6%)

## Data Availability

The data presented in this study are available on request from the corresponding author. These data are not publicly available because participants were not informed their raw data would be shared.
